# Contribution of Mg-templated porosity to activity and durability in Fe–N–C O_2_ reduction catalysts

**DOI:** 10.1039/d5ma01488c

**Published:** 2026-03-16

**Authors:** Angus Pedersen, Jinjie Zhu, Jesús Barrio, Joseph Parker, Robert D. Hunter, Sarah J. Haigh, Tim-Patrick Fellinger, Ifan E. L. Stephens, Maria-Magdalena Titirici

**Affiliations:** a Department of Materials, Imperial College London London SW7 2AZ UK angus-nils.pedersen@bam.de; b Department of Chemical Engineering, Imperial College London London SW7 2AZ UK j.barrio-hermida@imperial.ac.uk,m.titirici@imperial.ac.uk; c Division 3.6, Electrochemical Energy Materials, Bundesanstalt für Material-forschung und -prüfung (BAM), Unter den Eichen 44-46 12203 Berlin Germany; d Department of Materials, University of Manchester Manchester M13 9PL England; e Advanced Institute for Materials Research (WPI-AIMR), Tohoku University, 2-1-1 Katahira, Aobaku Sendai Miyagi 980-8577 Japan

## Abstract

Atomically dispersed Fe in N-doped carbon (Fe–N–C) catalysts are leading platinum-group-metal-free candidates for the O_2_ reduction reaction in proton exchange membrane fuel cells (PEMFCs). Zeolitic imidazolate framework (ZIF-8) derived Fe–N–C present the most promising performance; however, they possess a narrow distribution of small micropores, which limits active site accessibility. Here, to induce hierarchical porosity in Fe–N–C, we report a systematic study on MgCl_2_·6H_2_O-templated ZIF-8-derived Fe–N–C catalysts for the O_2_ reduction reaction. MgCl_2_·6H_2_O addition induced complete Zn removal, collapse of the ZIF-8 framework, and formation of large micro- and mesopores, with graphene-like structures. N content was markedly reduced, with conversion from pyridinic to pyrrolic N species. Rotating disc electrode tests showed a progressive increase in O_2_ reduction activity with MgCl_2_·6H_2_O, which is strongly correlated (*R*^2^ = 0.98) to the formation of large micropores and small mesopores (1–4 nm). This introduces an indirect structure–activity design principle for Fe–N–Cs. The enhanced Fe–N–C porosity also leads to increased degradation rates under accelerated stress test conditions, which we attributed to the oxidation of disordered carbon domains and active Fe loss. This study highlights a key trade-off between porosity-driven O_2_ reduction activity and durability in Fe–N–C catalysts.

## Abstract 2

The formation of Mg-templated pores within 1–4 nm strongly correlates with activity and degradation of Fe–N–C catalysts for the acidic O_2_ reduction reaction.

## Graphical abstract

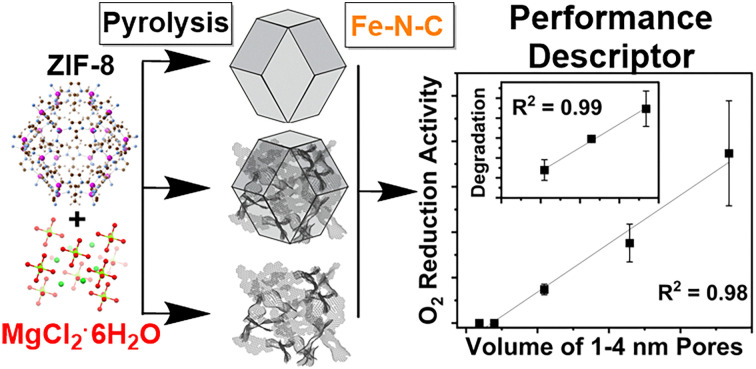

## Introduction

Low temperature proton exchange membrane fuel cells (PEMFCs) offer an efficient and zero CO_2_ emission operation technology for transport and back-up power applications. One limitation of PEMFCs is the significant use of expensive precious metals, such as Pt, at the cathode, where the O_2_ reduction reaction occurs.^1^ The next-generation PEMFCs are reducing Pt content based on improved catalyst and cell designs.^2^ Still, techno-economic projects of Pt nanoparticle on C (Pt/C) based cathodes in PEMFC at 500 000 stacks year^−1^ predict the Pt catalyst would make up 26% of the stack cost, based on US DOE 2025 Pt loading targets.^3^ Additionally, Pt is highly susceptible to poisoning from contaminants, such as CO, H_2_S and SO_2_.^4,5^ As an alternative, single metal atoms in N-doped C catalysts (M–N–C), in particular Fe–N–C, show the highest non-precious metal O_2_ reduction activity, and exhibit high poison tolerance.^6^

Among M–N–C precursors, zeolitic imidazolate framework (ZIF), such as ZIF-8, have been pivotal to PEMFC performance progress and scalability.^7–10^ ZIFs were first demonstrated by Yaghi and coworkers to present exceptional chemical and thermal stability compared to most metal organic frameworks.^11^ Additionally, many different ZIF topologies and structures are synthesizable,^12,13^ allowing the development of structure–activity descriptors, including the cavity size of the pristine ZIF.^14^ The high content of retained Zn–N_4_ sites^15,16^ and carbonaceous microporous structure of ZIF-8 following pyrolysis^17^ provides a suitable conductive template for O_2_ reduction active FeN_*x*_ (*x* = 1–5) sites.^18^ Fellinger and coworkers first introduced a Zn-imprinting and ion-exchange synthesis to decouple active site formation from pyrolysis, allowing low-temperature metalation and preventing undesired pyrolysis-driven carbide formation.^19–21^ These transmetalation routes have achieved record FeN_*x*_ active site densities and O_2_ reduction activity.^22,23^ Despite these advances, typically <10% of the FeN_*x*_ sites are electrochemically utilised (based on five electron *in situ* nitrite stripping), limiting the accessible FeN_*x*_ site density required to compete with Pt-based catalyst O_2_ reduction activity.^23,24^ This is due to the dominant microporosity of bulky ZIF-8-derived particles.^16,25^ Nonetheless, studies demonstrating clear correlations of porosity *versus* activity of M–N–Cs remain limited.^14,26,27^ Salt-templating with ZnCl_2_ or NaCl have demonstrated hierarchical porosity and opening of bottleneck pores to improve O_2_ reduction performance^26–31^ More recently, Mg-based templating has proven particularly effective.^32–38^ In the case of MgCl_2_·6H_2_O this was due to the generation of transient porogens^39^ and MgN_*x*_ active site templating.^32^ Using this approach, our group reported MgCl_2_·6H_2_O templated Fe–N–C with record electrochemical active utilisation of 52% (based on five electron *in situ* nitrite stripping),^37^ and up to 29 ± 4% in PEMFC (based on *in situ* Fourier transformed alternating current voltammetry).^24^ Addition of MgCl_2_·6H_2_O to ZIF-8 and their subsequent pyrolysis has been explored for Na-ion anodes to generate microporosity.^40^ It was found that due to the salt melt, the ZIF-8 structure dissolves during the carbonisation process, creating carbon nanosheets. This led to near complete removal of Zn, from 10 wt% in directly pyrolysed ZIF-8 to <0.3 wt% in Mg-doped samples.^40^

Aside from activity, stability of Fe–N–C under acidic conditions is challenging and insufficient, due to a combination of Fe dissolution, reactive oxygen species and carbon corrosion.^41–44^ So far, high surface area Mg-templated Fe–N–C have displayed fast degradation and poor active site stability under PEMFC-relevant conditions.^45,46^ Recently, Wu and coworkers reported trace Mg- doping (0.5% Mg) in ZIF-derived Fe–N–C can significantly improve the catalyst stability by altering the local FeN_*x*_ carbon structure, eliminating undesired N species and suppressing H_2_O_2_ formation.^47^

Here, we investigate how the Mg salt (MgCl_2_·6H_2_O) to ZIF-8 ratio controls the chemistry and morphology of ZIF-derived Fe–N–Cs and correlate porosity effects on activity and stability for O_2_ reduction in rotating disc electrode measurements.

## Experimental

**Fig. 1:**
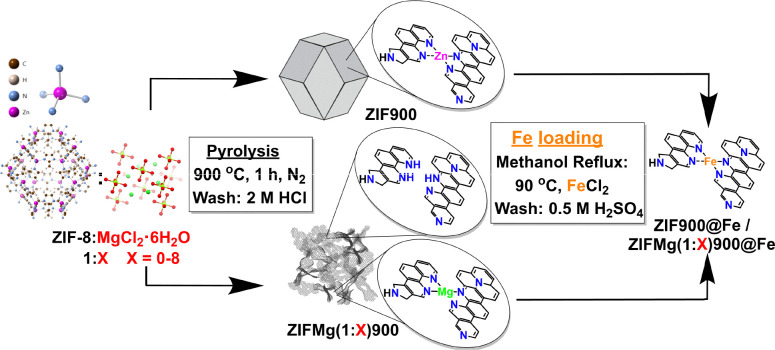
Schematic representation of the synthesis of ZIF-Mg-derived Fe–N–C catalysts.

The catalysts were synthesised based on Fig. 1, with the steps detailed in the SI. Briefly, commercial ZIF-8 (Basolite® Z1200) was either directly pyrolysed or mixed with MgCl_2_·6H_2_O (1 : 1–8 wt. ratio) and then pyrolysed (5 °C min^−1^) under N_2_ flow to 900 °C for 1 h. The resulting powders were ground, washed with 2 M HCl and then dried at 80 °C. The catalyst was then impregnated with Fe using FeCl_2_ under methanol reflux for 24 h and then washed with 0.5 M H_2_SO_4_, followed by 80 °C drying. Characterisation and electrochemical testing protocols are described in the SI.

## Results and discussion

### Catalyst characterisation

Pyrolysis of ZIF-8 at 900 °C and subsequent acid washing yielded 29% ZIF900 (Table S1), while addition of MgCl_2_·6H_2_O reducing the yield to 14–17%, indicating salt-mediated volatilisation during carbonisation. Post pyrolysis, ZIFMg(1 : 1)900 substantially increased in volume (Fig. S1a), whereas higher salt ratios produced denser products (Fig. S1b and c). As previously established,^32,33,37^ these differences arise likely due to the different templating pathways. At low Mg ratios, partial wetting of ZIF-8 produced a viscous matrix that trapped evolved gases and caused foaming, whereas at high Mg loadings, ZIF-8 dispersed in a molten salt phase that enabled gas release, yielding compact carbons. Thermal gravimetric analysis mass spectrometry (TGA–MS) confirmed distinct decomposition behaviour with increasing MgCl_2_·6H_2_O, shifting from oxidative carbon loss to chloride-driven dehydrogenation (Note S1 and Fig. S2).

Scanning electron microscopy (SEM) reveals the ZIF-8 structure is lost after MgCl_2_·6H_2_O addition (ZIFMg(1 : 1)900), forming a heterogeneous mixture of smooth, bubble-like particle sizes up to 50 µm (Fig. S3b), arising from the dominant bubble templating. Increasing MgCl_2_·6H_2_O content further (ZIFMg(1 : 2)900, Fig. S3c), the particles appear less well-defined, and a more porous structure develops as ZIF-8 is soaked by the salt. This trend continues with ZIFMg(1 : 4)900 and ZIFMg(1 : 8)900, with sheet-like open structures with lateral dimensions of up to ∼5 µm (Fig. S3d and e). Overlayed Raman spectroscopy and its deconvoluted peaks (Fig. S4) shows a distinct contribution of *D*3- (∼ 1495 cm^−1^) is present in ZIF-900 and ZIFMg(1 : 1)900. This is attributed to amorphous carbon species.^48^ This feature largely diminishes for higher ratios of MgCl_2_·6H_2_O, as demonstrated by the reduction in *I*_D3_/*I*_G_ (Table S2). Meanwhile, the *I*_D_/*I*_G_ ratio does not significantly change, indicating comparable lateral size of nano-crystallites. However, the full-width half maximum of *D* and *G* bands (*ca.* 1585 cm^−1^, arising from quasi-graphitic crystallites) significantly decreases. The reduction in *D*3 band intensity upon adding MgCl_2_·6H_2_O, indicates less disordered sp^2^–sp^3^ carbon domains. This may be due to facilitated conversion of aliphatic intermediates due to increased reorganizational dynamics in the presence of the molten salt. X-ray diffraction (XRD) confirms collapse of ZIF-8 crystallinity with pyrolysis, and disappearance of C (002) reflection at 2-theta of ∼25° with MgCl_2_·6H_2_O addition (Fig. S5), indicating atomically thin graphene-like structure with limited long-range carbon stacking.

**Fig. 2:**
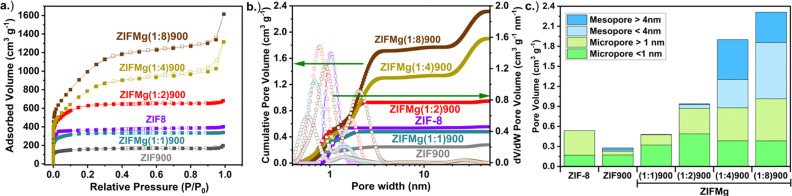
(a) N_2_ sorption isotherms and (b) corresponding pore size distributions based on heterogeneous surface 2D non-linear density functional theory (HS 2D NLDFT) model. (c) Pore volumes at defined pore ranges.

N_2_ sorption of as received ZIF-8 presents a Brunauer–Emmett–Teller specific surface area (*S*_BET_) of 1170 m^2^ g^−1^ (Fig. 2a and Table S3), which is comparable to previous reports.^49^ Upon pyrolysis, ZIF-900 shows a reduced *S*_BET_ of 568 m^2^ g^−1^. Mehmood *et al.* showed the ZIF-8 specific surface area can largely be recovered post pyrolysis by their activation and dicyandiamide doping protocol of the Fe–N–C, reaching 1155 m^2^ g^−1^. The cumulative pore volume up to a pore width of 4 nm increased 60%, with the electrochemical active site density increasing more than fourfold.^23^ Here, it was found the starting *S*_BET_ of ZIF-8 could be retained with ZIFMg(1 : 1)900, at 1138 m^2^ g^−1^. As expected, increasing the MgCl_2_·6H_2_O ratios led to increased *S*_BET_, up to 3548 m^2^ g^−1^ at ZIFMg(1 : 8)900, comparable to our previously reported Mg-templated carbons.^45,50^

The corresponding pore size distributions from the heterogeneous surface 2D non-linear density functional theory (HS 2D NLDFT) model reveal that ZIF-8 is purely microporous with pore diameters centered at 1 nm, which contracts to 0.5 nm after pyrolysis (Fig. 2b). Increasing MgCl_2_·6H_2_O progressively widens the micropores, reaching 0.9 nm at ZIFMg(1 : 4)900. The introduction of additional pore diameter at 2 nm and above 40 nm becomes pronounced. The sub-defined micro- and mesopore volume values are displayed in Fig. 2c and Table S3, showcasing how micropore volumes increase up to 0.88 cm^3^ g^−1^ in ZIFMg(1 : 2)900 and then plateau, meanwhile mesopore volumes increase up to 0.58 cm^3^ g^−1^ in ZIFMg(1 : 4)900 and then plateaus. A similar evolution was found by Pampel *et al.* when varying the MgCl_2_·6H_2_O ratio with adenine-derived carbons, although in their case a more tubular N–C porous structure was created due to the self-assembly of adenine and its polycondensate products with the water molecules within the Mg salt.^33^

Inductively coupled plasma mass spectrometry (ICP-MS, Fig. S6a) on commercial ZIF-8, as received, contained 28.29 ± 0.70 wt% Zn, decreasing to 8.71 ± 0.65 wt% after pyrolysis (ZIF900). In ZIFMg(1 : 1)900 (1.12 molar ratio for MgCl_2_·6H_2_O:ZIF-8), Zn reduced to 0.46 wt% with 1.51 wt% Mg. ZIFMg(1 : 2)900 contained only 0.1 ± 0.01 wt% Zn and 0.72 ± 0.04 wt% Mg, while only trace amounts of metals remain in ZIFMg(1 : 4)900, consistent with Zn volatilisation (Zn boiling point around 918 °C)^15^ and complete Mg template removal. This is consistent with Mehmood *et al.* who observed removal of the ZIF-8 structure and Zn removal upon addition of high wt. ratio of MgCl_2_·6H_2_O (1 : 10).^40^ This is likely caused by ZIF-8 dissolution in the liquid phase and Mg salt-melt breaking the Zn coordination to 2-methyl-imidazole.

Fe introduced *via* low temperature methanol reflux showed sample-dependent incorporation. Mehmood *et al.* achieved up to 7.1 wt% Fe loading with only 1.0 wt% of Zn remaining by carrying out activation steps on a carbonised ZIF-8 (Fig. S6b).^23^ The stronger binding energy of FeN_4_ compared to ZnN_4_ allows thermodynamically favourable ion exchange, with kinetically feasible activation barriers.^51^ Meanwhile, directly loading Fe into ZIF900 (ZIF900@Fe) leads to 1.00 ± 0.13 wt% of Fe and reduction of Zn to 7.85 ± 0.27 wt%. In contrast, ZIFMg(1 : 1)900@Fe contained 0.3 wt% Fe and 0.25 wt% Zn, and Mg unchanged (1.50 wt%). The reciprocal changes in Fe and Zn content indicate preferential Fe–Zn transmetalation, rather than Fe–Mg exchange. Meanwhile, only 0.18 wt% Fe is present in ZIFMg(1 : 2)900@Fe and Mg dropped to 0.03 wt%, with Zn remaining equivalent (0.08 wt%). This suggests Fe may have transmetalated with Mg in MgN_*x*_ sites or loaded into empty N_*x*_ sites. On the other hand, the increase in Fe content in ZIFMg(1 : 4)900@Fe and ZIFMg(1 : 8)900@Fe likely reflects Fe metalation into empty N_*x*_ sites.

XRD patterns of Fe-loaded catalysts show identical carbon reflections to Mg-templated NC, confirming the absence of large or concentrated crystalline Fe phases (Fig. S7).

**Fig. 3:**
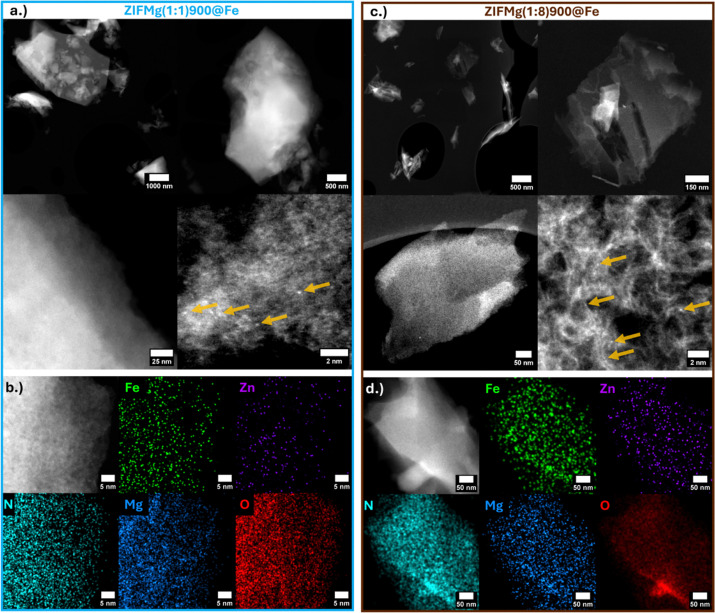
(a) HAADF-STEM of ZIFMg(1 : 1)900@Fe and (b) HAADF-STEM with EDXS mapping. (c) HAADF-STEM of ZIFMg(1 : 8)900@Fe and (d) HAADF-STEM with EDXS mapping. Locations of bright spots (single atoms) are highlighted by yellow arrows.

High angle annular dark field-scanning transmission electron microscopy (HAADF-STEM) imaging of ZIFMg(1 : 1)900@Fe reveals partially retained ZIF-8 morphology (Fig. 3a), with isolated bright atomic features attributed to Fe or residual Zn, rather than Mg, due to their higher atomic mass. Elemental mapping demonstrates a uniform dispersion of Fe, Zn and Mg, with no evidence of nanoparticle formation. (Fig. 3b and Fig. S8a, S9b, c). In ZIFMg(1 : 8)900@Fe, large micro- and mesopores are clearly visible (Fig. 3c), consistent with N_2_ sorption data (Fig. 2b). Atomic metal dispersion is also evident in the largely exfoliated graphene-like layers (Fig. 3c and d), showing nm-sized graphitic carbon domains, analogous to previous Mg-templated N–C.^24^ Elemental mapping show the even dispersion of elements, with energy dispersive X-ray spectroscopy showing a weak but detectable Fe signal, with little to no Zn or Mg remaining (Fig. S8d). Based on ICP-MS (Fig. S7) and STEM-EDXS, the identified single atoms are therefore expected to be predominantly Fe. Due to the low Fe loading in Mg-templated Fe–N–Cs here (≤0.3 wt% Fe), clear characterisation on the FeN_*x*_ active sites beyond HAADF-STEM and STEM-EDXS remains challenging. For insights into how FeN_*x*_ changes with Mg-templating we refer the reader to our recent works.^37,38,52^ Here, focus is made on the clearly distinguishable catalyst properties on performance, such as porosity and N type.

**Fig. 4:**
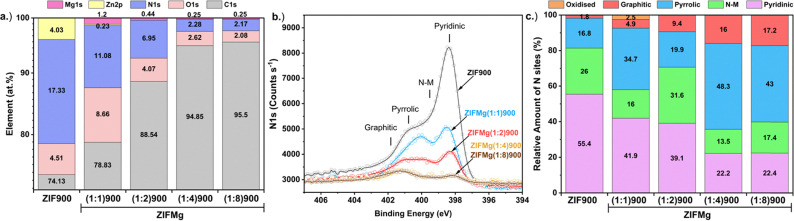
XPS of ZIF-8 derived and Mg-doped catalysts. (a) Elemental composition. (b) Overlays of N1s and (c) corresponding fitted N species composition.

Overlaid XPS spectra of C1s, O1s, N1s, Zn2p and Mg1s of ZIF900 and ZIFMg(1 : X)900 catalysts are shown in Fig. S9 with the corresponding at% element composition in Fig. 4a. The increased O content in ZIFMg(1 : 1)900 may correspond to MgO remaining after acid washing due to limited accessibility. With increasing Mg precursor ratio, the C content increases while O and Mg content decreases. The 4.0 at% of Zn in ZIF900 is almost entirely removed for ZIFMg(1 : 1)900, demonstrating the dissolution of the ZIF-8 structure.

The overlaid N1s spectra demonstrates the reduction in N species upon increasing MgCl_2_·6H_2_O ratios. Overlaid N1s spectra are shown in Fig. 4b with their quantities plotted in Fig. 4c and fittings displayed in Fig. S10. N content falls from 17.3 at% in ZIF900 to 2.2 at% in ZIFMg(1 : 8)900. Meanwhile, increasing MgCl_2_·6H_2_O results in a relative increase in graphitic N, from 1.8 to 17.2% between ZIF900 and ZIFMg(1 : 8)900, respectively. Additionally, a clear trend of relative decreasing pyridinic N content and increasing pyrrolic N is found with increasing MgCl_2_·6H_2_O. According to density functional theory studies and recent X-ray absorption spectroscopy observations, this could be due to Mg having a higher affinity towards pyrrolic motifs than Zn, which could lead to greater pyrrolic retainment.^52,53^ Elemental analysis also confirms a sharp decrease in N content from 15.2 to 2.3 wt% with increasing MgCl_2_·6H_2_O precursor, indicating comparable bulk and surface composition (Fig. S11).

Cryogenic (5 K) *X*-band electron paramagnetic resonance (EPR) reveals suppression of carbon-centred radicals upon Mg addition (*g* ∼ 2) and predominantly EPR-inactive Fe^2+^ species after Fe loading (Fig. S12), which is regarded as the less active but more durable FeN_*x*_ site for O_2_ reduction.^54^

### Correlating with O_2_ reduction performance

**Fig. 5:**
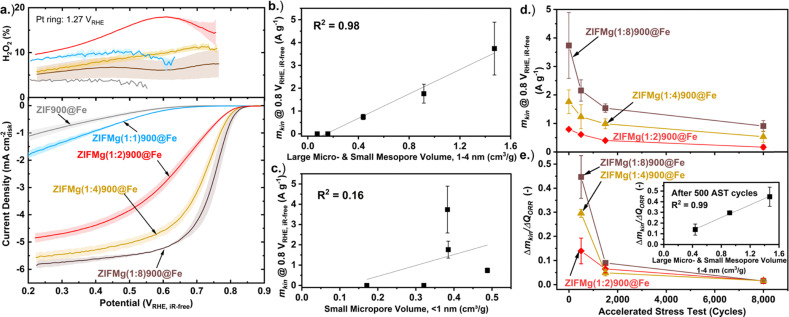
(a) 3rd cathodic scan of cyclic voltammogram with RRDE at 1600 rpm in O_2_-saturated 0.1 M HClO_4_ from 0.90–0.20 V_RHE_ at 10 mV s^−1^ with 0.26 mg_Fe–N–C_ cm^−2^. Measurements under equivalent N_2_-saturated conditions were subtracted from those measured under O_2_-saturation. Error bars are shown in shaded regions, which represent the error of two measurements carried out on separate catalyst inks. Correlation between kinetic mass activity at 0.8 V_RHE,iR-free_ and (b) large micro- and small mesopore volume, 1–4 nm. (c) Small micropore volume, <1 nm. (d) Kinetic mass activity (*m*_kin_) over accelerated stress test (AST: cycling 0.80–0.40 V_RHE_ at 100 mV s^−1^ under O_2_-saturation at 1600 rpm). (e) Degradation rate represented by change in *m*_kin_ normalised to the change in charge passed over AST cycles, with inset showing correlation to large micro- and small mesopore volume values after 500 AST cycles.

The O_2_ reduction performance of the catalysts was tested in O_2_-saturated 0.1 M HClO_4_ three-electrode rotating ring disc electrode (RRDE) setup under 1600 rpm (0.26 mg_M–N–C_ cm^−2^ loading). Prior to Fe loading, all ZIF-derived catalyst displayed negligible O_2_ reduction activity, only beginning to show current onset (>0.1 mA cm^−2^) below 0.6 V_RHE_ (Fig. S13). Following Fe incorporation, ZIF900@Fe displayed H_2_O_2_ production <5% across the potential range; however, the disk current density did not exceed 1.25 mA cm^−2^, despite high Fe content (1.0 wt%, Fig. S6b), suggesting limited FeN_*x*_ accessibility. The H_2_O_2_% decreases from ZIFMg(1 : 2)900@Fe to ZIFMg(1 : 8)900@Fe (Fig. 5a and eqn (S4)). This improvement likely stems from enhanced porosity and surface area, enabling secondary reduction of H_2_O_2_ within the catalyst layer,^55^ and/or increased electrochemical accessibility of FeN_*x*_ active sites.^37^

Increasing the MgCl_2_·6H_2_O precursor ratio leads to improved O_2_ reduction activity, particularly for Mg-templated samples with higher Mg precursor ratios (*X* ≥ 2, Fig. 5), despite comparable Fe loadings (0.18–0.30 wt%, Fig. S7). This correlation is strongest (*R*^2^ = 0.98) when considering the increasing volume of large micropores and small mesopores (1–4 nm, Fig. 2c) with the kinetic mass activity (*m*_kin_, eqn (S2)) at 0.8 V_RHE,iR-free_ (Fig. 5b). This follows observations by Wang *et al.* where pores between 0.8–2.0 nm contributed the majority of Fe–N–C O_2_ reduction activity.^27^ Additionally, Wan *et al.* reported mesopore incorporation into ZIF-8-derived Fe–N–C increased FeN_*x*_ electrochemical utilisation and site density, leading to improved PEMFC performance.^30^ We relate these combined findings to FeN_*x*_ active sites becoming electrochemically accessible above specific pore diameters. We note that porosity has an indirect effect on activity, rather than direct changes in the intrinsic activity of FeN_*x*_ sites. Consequently, porosity can be considered as a secondary (indirect), rather than primary, structure–activity descriptor. No correlation is observed when considering small micropores <1 nm (*R*^2^ = 0.16, Fig. 5c), indicating small micropores do not contribute to O_2_ reduction activity. The correlation of other pore volumes with activity is shown in Fig. S14.

We note that our previously Mg-templated Fe–N–C presented a low intrinsic O_2_ reduction activity (turnover frequency) compared to the state-of-the-art.^37^ This also likely restricts the measured initial kinetic mass activity here (Fig. 5d), in addition to the limited site density from the low Fe loading (Fig. S6b).

Accelerated stress testing (AST; 0.80-0.40 V_RHE_, 100 mV s^−1^ O_2_-saturated, 1600 rpm; Fig. 5d) shows that while ZIFMg(1 : 8)900@Fe exhibits the highest initial kinetic mass activity (3.74 ± 1.14 A g_Fe–N–C_^−1^), it undergoes the fastest degradation. After 8000 AST cycles all electrocatalysts displayed H_2_O_2_ yield >20% across the potential range (Fig. S15). When normalised to the total O_2_ reduction charge (*Q*_ORR_, eqn (S5)), degradation scales with the volume of 1–4 nm pores (inset, Fig. 5e). This shows the most active regions of the Fe–N–Cs are also the most unstable. After 8000 AST cycles the rate of degradation converge and become comparable. This may due to a shift of degradation mechanism from initially primarily Fe dissolution to reactive oxygen species attack and carbon corrosion,^41,42,44^ as we previously resolved for another Mg-templated Fe–N–C.^45,46^ It is also noted there is no observable stabilisation effect from Mg doping, unlike in the work by Wu and coworkers, where Mg acetylacetonate was added during ZIF-8 synthesis which led to elimination of undesired N-species in their Fe–N–C.

Post-AST HAADF-STEM of ZIFMg(1 : 8)900@Fe shows retained morphology and some single atoms remaining (Fig. S16), with EDXS demonstrating reduced Fe signal (Fig. S17 *versus* Fig. S8), indicating partial removal of active Fe single atoms. The rapid Fe and activity loss in highly porous Mg-templated samples aligns with our prior reports of rapid dissolution from exposed FeN_*x*_ sites, as confirmed by online ICP-MS in both flow cell and gas diffusion electrode setups.^46^ Moreover, Fig. S9a demonstrates an increase in C–O, C=O and C_*x*_Z_*y*_O_w_ species with increasing Mg precursor. Based on previous post-mortem Raman and temperature programmed desorption on equivalently Mg-templated N–Cs,^45,56^ carbon oxidation likely further contributes to instability and reduced turnover frequency,^42^ as amorphous and defective graphitic domains (Fig. S4) are preferentially corroded within the 0.8–0.4 V_RHE_.^57^ N_2_-saturated cyclic voltammograms confirms this: only ZIFMg(1 : 8)900@Fe displays the clear emergence of ill-defined redox, indicative of quinone/hydroquinone species resulting from carbon oxidation (Fig. S18).^58^ In summary, this is indicative that the Mg-templating process, which generates porosity under these conditions, yields N–C (and subsequent Fe–N–C) that are oxidised and prone to oxidation and corrosion within the applied potential range used for the AST. Consequently, unlike Mg acetylacetonate doping during ZIF synthesis reported by Wu *et al.*,^47^ no Mg stabilisation effect is observed here. This underscores the timing, type and mechanism of Mg introduction critically determine the balance between activity and durability in Fe–N–C catalysts.

## Conclusions

Increasing MgCl_2_·6H_2_O precursor with ZIF-8 led to atomically thin carbon sheets, and reduced N content, shifting from predominantly pyridinic to pyrrolic N species. Clear increases in large micropores and mesopores were observed, which correlated strongly with improved electrocatalytic activity, culminating in a peak kinetic mass activity of 3.74 ± 1.14 A g^−1^ at 0.80 V_RHE,iR-free_ for ZIFMg(1 : 8)900@Fe. Despite the high activity and low peroxide yield, this catalyst displayed the most significant degradation during the AST. This is related to porosity *via* increased (1) risk of Fe dissolution from increased exposure and (2) amounts of oxidised and oxidisable carbon species from Mg-templating. This reveals the multifaceted role of porosity on electrocatalyst performance.

## Author contributions

A. P. conceptualised the work and wrote the initial draft and led the investigation. A. P. synthesised the materials and performed XRD, Raman, N_2_ sorption, EPR, ICP-MS, and electrochemical testing and their analysis. J. Z. performed SEM measurements. R. H. provided TGA-MS measurements and Raman analysis. J. P. performed HAADF-STEM and EDXS. J. B. carried out XPS measurements and analysis. J. B., S. J. H., M.-M. T., and I. E. L. S. provided project supervision, and funded the project and resources. All authors contributed to reviewing and editing the manuscript.

## Conflicts of interest

There are no conflicts of interest to declare.

## Supplementary Material

MA-007-D5MA01488C-s001

## Data Availability

Experimental data files from the manuscript are available at BAM Publica: https://doi.org/10.26272/opus4-65228. Supplementary information (SI) is available. See DOI: https://doi.org/10.1039/d5ma01488c.
