# Supplementary material for: Contribution of Mg-templated porosity to activity and durability in Fe–N–C O2 reduction catalysts
Source: Mater Adv. 2026 Mar 16;7(7):3524–31. doi: 10.1039/d5ma01488c (PMC12990056; doi:10.1039/d5ma01488c)
Supplement: MA-007-D5MA01488C-s001 [file MA-007-D5MA01488C-s001.pdf]

Supplementary Information (SI) for Materials Advances.
This journal is © The Royal Society of Chemistry 2026

Supporting Information:

Contribution of Mg-templated Porosity to Activity and Durability in Fe-N-C O_2 Reduction Electrocatalysts

Angus Pedersen,[a,b,c,]* Jinjie Zhu,[b] Jesús Barrio,[b,]* Joseph Parker,[d] Robert D. Hunter,[b] Sarah J. Haigh,[d] Tim-Patrick Fellinger,[c] Ifan E. L. Stephens,[a] Maria-Magdalena Titirici,*[,b,e]

a) *Department of Materials, Royal School of Mines, Imperial College London, London SW7 2AZ, England.*
b) *Department of Chemical Engineering, Imperial College London, London SW7 2AZ, England.*
c) *Division 3.6 Electrochemical Energy Materials, Bundesanstalt für Materialforschung und -prüfung (BAM), Unter den Eichen 44-46, 12203 Berlin, Deutschland, Germany*
d) *Department of Materials, University of Manchester, Manchester, M13 9PL, England.*
e) *Advanced Institute for Materials Research (WPI-AIMR), Tohoku University, 2-1-1 Katahira, Aobaku, Sendai, Miyagi, 980-8577, Japan.*

* Corresponding authors:

Experimental

Synthesis of Fe-N-C: ZIFMg(1:X)900 was prepared by grinding commercial ZIF-8 (Basolite® Z1200, produced by BASF) with magnesium chloride hexahydrate (99%, Sigma Aldrich) with a pestle and mortar in a 1:X weight ratio, where X = 0, 1, 2, 4 or 8. The mixture was then placed in an alumina crucible with a lid in an alumina furnace tube (Ø = 50 mm) and pyrolysed under N_2 (300 mL min^{-1}, >99.998%, BOC) from room temperature to 900 °C at a heating rate of 5 °C min^{-1}, holding at 900 °C for 1 h, before cooling down naturally. The pyrolysed mixture was then ground down to a powder with a pestle and mortar and washed in 2 M HCl (prepared by dilution of fuming 37% HCl, Merck) overnight. The solution was then filtered and rinsed thoroughly with DI water and dried at 80 °C in an oven overnight, ZIFMg(1:X)900. Fe was coordinated by a low-temperature wet impregnation methanol reflux.[1–3] Here, 60 mg of ZIFMg(1:X)900 was placed in a 250 mL round bottom flask with 75 mL MeOH (AnalaR NORMAPUR Reag. Ph. Eur., ACS, VWR) under magnetic stirring until a homogeneous dispersion was observed. Then, 75 mL of $FeCl_2$ (98% Sigma Aldrich) solution in MeOH (25 × 10^{-3} M) was added to the ZIFMg(1:X)900 MeOH solution and the mixture was subjected to reflux at 90 °C for 24 h. The solution was filtered with MeOH, and the dried powder was washed in 0.5 M H_2SO_4 (prepared from 95-98% Sigma Aldrich) overnight. The solution was then filtered and washed thoroughly with DI water and dried in an oven at 80 °C, creating ZIFMg(1:X)900@Fe.

HAADF-STEM and EDXS: The morphology and chemical composition of ZIFMg(1:X)900@Fe was characterized by scanning transmission electron microscopy (STEM) and energy dispersive X-ray spectroscopy (EDXS) using an FEI Titan G2 80-200 S/TEM ChemiSTEM microscope equipped with an X-FEG high brightness source and a STEM probe aberration corrector. The microscope was operated with an accelerating voltage of 200 kV and a beam current of 100 pA beam current. The probe convergence angle was 21 mrad, and the HAADF detector inner collection angle was 84 mrad. EDXS spectrum imaging used Thermo Fisher Velox control software and a Super-X detection system with four silicon drift detectors and a total collection solid angle of approximately 0.7 sr. TEM samples were prepared by dusting dry catalyst powder onto a holey carbon-coated Cu support grid.

TGA-MS: Thermogravimetric analysis was performed using a Mettler Toledo TGA instrument coupled with a Hiden HPR-20 mass spectrometer using bar scan mode measuring in 1-27 Da and 29-300 Da

range. Sample was heated in a platinum pan from 25 to 100 °C at a rate of 10 °C/min and held for 30 min to drive off moisture. Sample was then further heated to 900 °C again using a heating rate of 10 °C/min.

SEM: SEM images were recorded on a Zeiss LEO Gemini 1525 scanning electron microscope (Carl Zeiss, Germany) at an accelerating voltage of 5 kV.

Raman: Raman spectra were obtained using an inVia Renishaw confocal Raman microscope operated with an incident LASER (Light Amplification by Stimulated Emission of Radiation) beam at 532 nm focused through a 50× objective (Leica), with 2.5 mW laser intensity, 10 s exposure time with the average of at least nine measurements taken. Spectra were normalised to the intensity of the graphitic lattice (*ca.* 1590 cm^{-1}), meaning that any change in the proportion of ordered/disordered carbon domains results in modified *D* band frequency/shape in the Raman spectra.[4] Nine repeat measurements were taken with each spectra deconvoluted using a four-peak model with pseudo-Voigt line shapes to extract average I_D/I_G values along with their standard deviation. The additional *D*3 and *D*4 bands used in the deconvolution procedure have been previously ascribed to amorphous carbon[5] and polyene structures,[6] respectively.

XRD: Samples were measured using a Malvern Panalytical Aeris X-ray diffractometer with a Cu Kα source operated at 40 kV n 30 mA with 0.022 ^{o} scan step size from 5 to 60 ^{o}.

N_2 sorption: N_2 sorption isotherms were conducted at -196°C in the pressure range from 10^{-5} to 0.99 using an Anton Paar Autosorb system. The samples were degassed overnight (16 h) at 250 °C. The BET specific surface area was obtained by taking the adsorption isotherm in the relative pressure range of <0.3 and the best region for linear fit of 10 points (R^2 > 0.995) as determined by the Rouquerol method.[7] The pore size distribution was obtained from the adsorption isotherm by heterogeneous surface carbon 2D-non-local density functional theory (NLDFT) method, which incorporates more realistic energetical heterogeneity and geometrical corrugation of the carbon surface and avoids artefacts.[8] The pore size distribution was plotted from 0.3-50.0 nm using SAIEUS software version 3.06.

EPR: Continuous wave electron paramagnetic resonance (EPR) was measured employing an X-band CW ELEXSYS E500 EPR spectrometer (Bruker, German) with a cryogen-free variable temperature cryostat (Oxford Instruments, Oxfordshire, UK). For such measurement, approximately 2 mg of material was added to a 3 mm Wilmad quartz (CFQ) EPR tube (Sigma Aldrich). The spectra were obtained at temperature of 5 K and microwave frequency of 9.707 GHz with 20 mW incident microwave power, 100 kHz modulation frequency, 1 G modulation amplitude, 10 dB power attenuation and with magnetic field sweeping in 0.5 Gauss increments from 50 to 5000 Gauss.

Elemental Analysis: ThermoFisher FlashSmart was used to detect C, N, H, and S, using V_2O_5 and 2,5-Bis(5-tert-butyl-benzoxazol-2-yl)thiophene are calibration standards. Samples were heated to 950 ^{o}C with 300 mL min^{-1} O_2 supplied. Sample measurements were repeated twice.

2.1 Synthesis

which is also described in the **Supporting Information**.

2.2 Characterisation

2.2.2 Electrochemical Testing

Electrochemical O_2 reduction experiments were conducted using an AUTOLAB PGSTAT302N potentiostat in a three-electrode configuration within a glass cell (Metrohm). 0.1 M $HClO_4$ electrolyte (TraceSELECT™ Ultra, Fluka™, Honeywell Chemicals) was saturated with either N_2 (>99.99998% BIP®

Plus, Air Products) or O_2 (>99.9998% Ultrapure Plus, Air Products). An Ag/$AgCl_{sat}$ (3 M KCl) electrode served as the reference, while a glassy carbon rod functioned as the counter electrode. The reference electrode was calibrated before each electrochemical test in a separate cell containing 0.1 M $HClO_4$, purged with hydrogen (1 bar) for 10 min. During calibration, a 3 mm diameter Pt RDE tip (Metrohm) was rotated at 1,600 rpm while five cyclic voltammograms were recorded at 10 mV s^{-1} between -0.26 and -0.28 $V_{Ag/AgCl}$. A Pt rod (Metrohm) and Ag/$AgCl_{sat}$ served as the counter and reference electrodes, respectively. The conversion between Ag/$AgCl_{sat}$ and reversible hydrogen electrode (RHE) was determined from the average potential of the forward and backward scans at zero current.

Post-experiment, iR compensation was applied by determining the solution resistance (23 Ω) from the intercept of the imaginary axis in the Nyquist plot, obtained via electrochemical impedance spectroscopy (10^{-5} – 10 Hz) at open circuit potential. The 5 mm diameter glassy carbon rotating disk electrode (RDE, Metrohm) was polished using a micropolish cloth and a 0.05 µm alumina suspension (Buehler) prior to each experiment.

Catalysts were drop-cast onto the freshly polished RDE from an ink prepared by dispersing 4 mg of catalyst in a mixture of 480 µL deionized water (18.2 MΩ cm), 480 µL isopropanol (≥99.5%, Honeywell™, Fisher Scientific), and 40 µL of 5 wt% Nafion® D-521 (Fuji Film). The ink was sonicated for 30 min in the centre of a bath sonicator (132 kHz, VWR) before deposition. A 12.7 µL aliquot was drop-cast onto the electrode and dried at 700 rpm for 30 min under ambient air, yielding a catalyst loading of 0.26 $mg_{M\text{-}N\text{-}C}$ cm^{-2}.

Cyclic voltammograms were recorded after purging the electrolyte for at least 15 min. Measurements were conducted at 50 mV s^{-1} with a stationary electrode (0 rpm) in N_2-saturated electrolyte (10th cycle displayed) and at 10 mV s^{-1} with a rotating electrode (1,600 rpm) in O_2- or N_2-saturated electrolyte (3rd cycle displayed). Pseudocapacitance was corrected by subtracting the N_2-saturated measurements at 1,600 rpm from the O_2-saturated results.

Electrochemical measurements were carried out in a RRDE to calculate the number of electrons transferred. The ink formulation and loading were identical to those used during RDE measurements. The Pt ring was set to 1.27 V_{RHE}. The number of electrons transferred (*n*) was calculated from the disk and the ring current using the following Equation S2:[9]

$$n = 4 \times \frac{j_d}{j_d + \frac{j_r}{N_c}} \qquad \textbf{Eq. S3}$$

Where j_r is the ring current and N_c is the collection efficiency (assumed as 21.67%).

Additionally, the H_2O_2% was obtained from Equation S4:

$$H_2O_2\,\% = 2 \times \frac{\frac{j_r}{N_c}}{j_d + \frac{j_r}{N_c}} \times 100 \qquad \textbf{Eq. S4}$$

Accelerated stress test (AST) cyclic voltammetry cycles were conducted between 0.80-0.40 V_{RHE} at 100 mV s^{-1} under O_2-saturation and 1,600 rpm.

The kinetic current density (j_{kin}) at potentials of 0.80 V_{RHE} was determined using the geometric disk current density (j_d) and geometric diffusion-limited current density (j_{lim}) at 0.25 V_{RHE}, employing the Koutecký–Levich Equation S1:

$$j_{kin} = \frac{j_d \times j_{lim}}{j_{lim} - j_d}$$ **Eq. S1**

Subsequently, the kinetic mass activity at 0.80 can be found:

$$m_{kin} = \frac{j_{kin}}{Loading_{FeNC}}$$ **Eq. S2**

Where m_{kin} is the kinetic mass activity at 0.80 V_{RHE} (A g_{FeNC}^{-1}) and $Loading_{FeNC}$ is the catalyst loading on the RDE (0.26 $mg_{Fe\text{-}N\text{-}C}$ cm^{-2}).

The amount of O_2 reduction charge passed over the AST (Q_{ORR}) was calculated based on the integration of the O_2 reduction current over time of the AST:

$$Q_{ORR} = \int I(t)dt$$ **Eq. S5**

Where I is the current (A).

Results

Table S1. Yield of synthesised catalysts prior to Fe loading. Yield based upon 1 g of ZIF-8 precursor.

Sample	Yield (%)
ZIF900	29.0
ZIFMg(1:1)900	16.8
ZIFMg(1:2)900	15.1
ZIFMg(1:4)900	17.0
ZIFMg(1:8)900	14.1

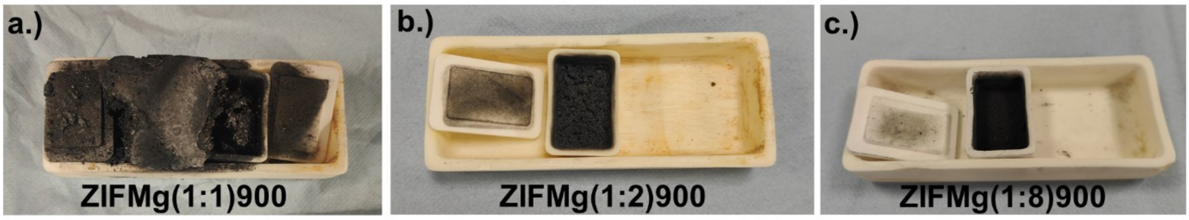

Figure S1. Photographs of Mg-doped ZIF-derived catalysts. a.) ZIFMg(1:1)900 b.) ZIFMg(1:2)900 and c.) ZIFMg(1:8)900 directly after pyrolysis (prior to grinding and acid washing).

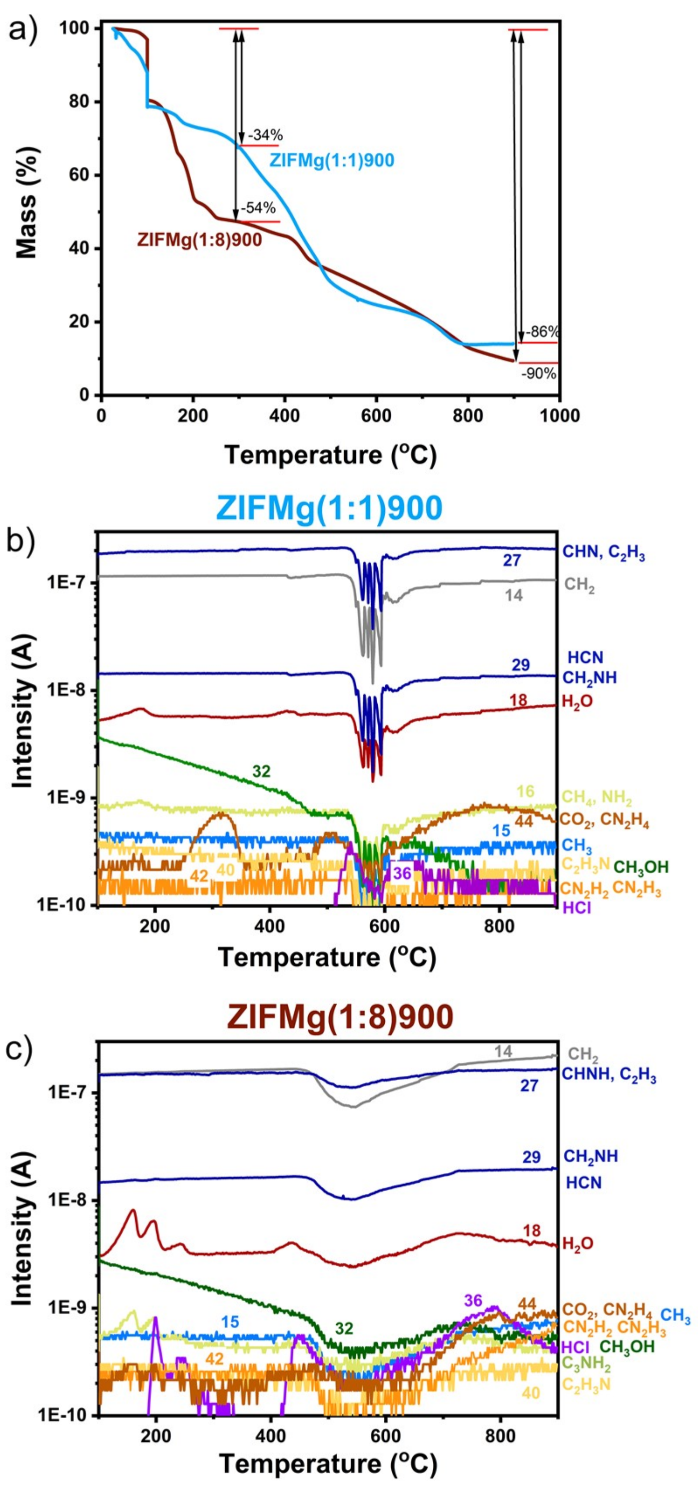

Figure S2. a.) TGA of ZIFMg(1:1)900 and ZIFMg(1:8)900 and MS on b.) ZIFMg(1:1)900 and c.) ZIFMg(1:8)900. Numbers on (b) and (c) refer to the mass detected.

Supporting Note 1:

TGA of ZIFMg(1:8)900 showed increased mass loss at 300 °C (54%) versus ZIFMg(1:1)900 (34%), which can be ascribed to the greater release of H_2O from $MgCl_2 \cdot 6H_2O$. CO_2 release is observed in ZIFMg(1:1)900 between 250-350 °C meanwhile HCl is released in ZIFMg(1:8)900 between 200-300 °C. The spikes in intensity around 550-600 °C in ZIFMg(1:1)900 could be related to bubbling from bubble templating.

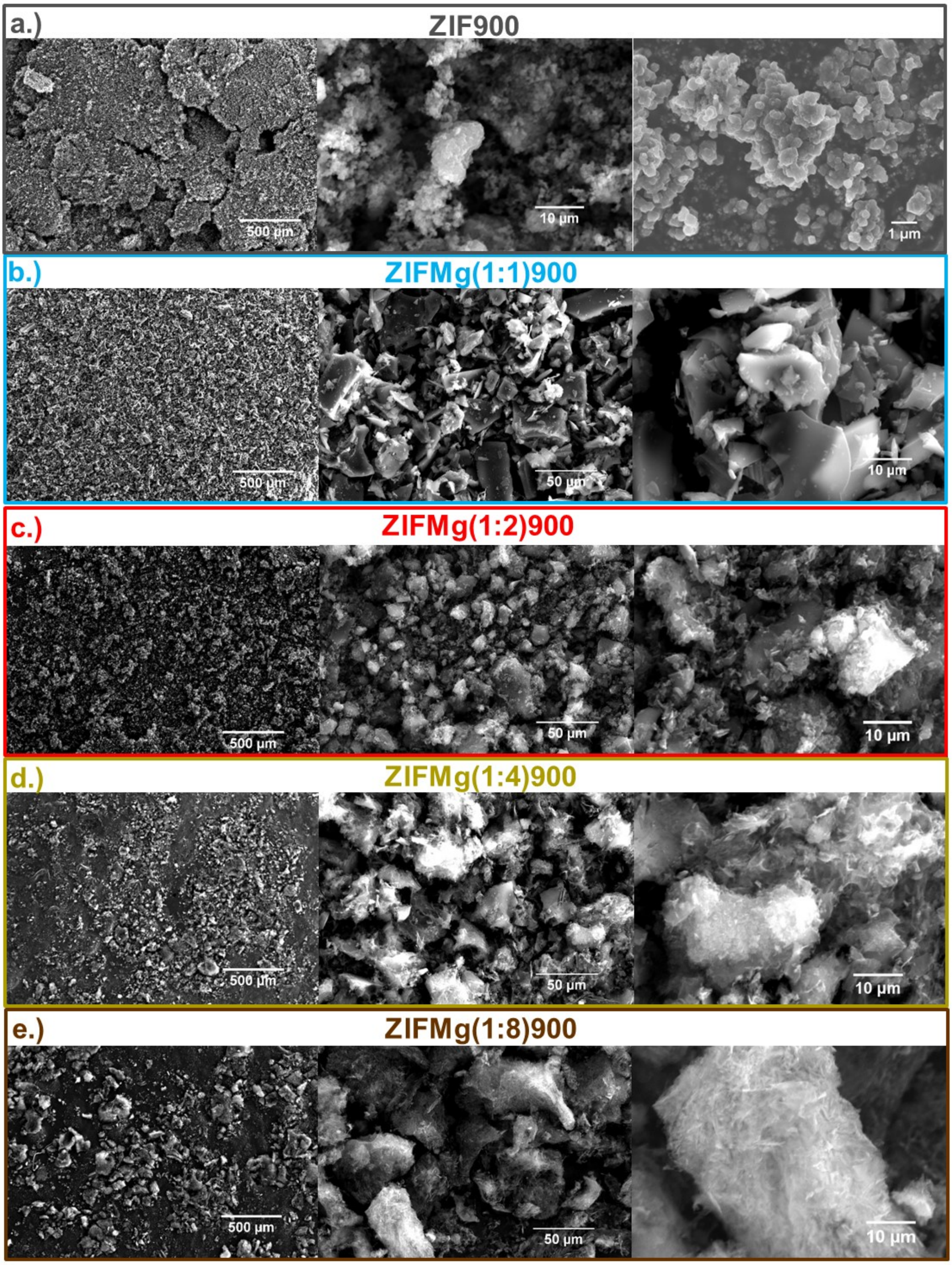

Figure S3. SEM of a.) ZIF900 b.) ZIFMg(1:1)900 c.) ZIFMg(1:2)900 d.) ZIFMg(1:4)900 e.) ZIFMg(1:8)900.

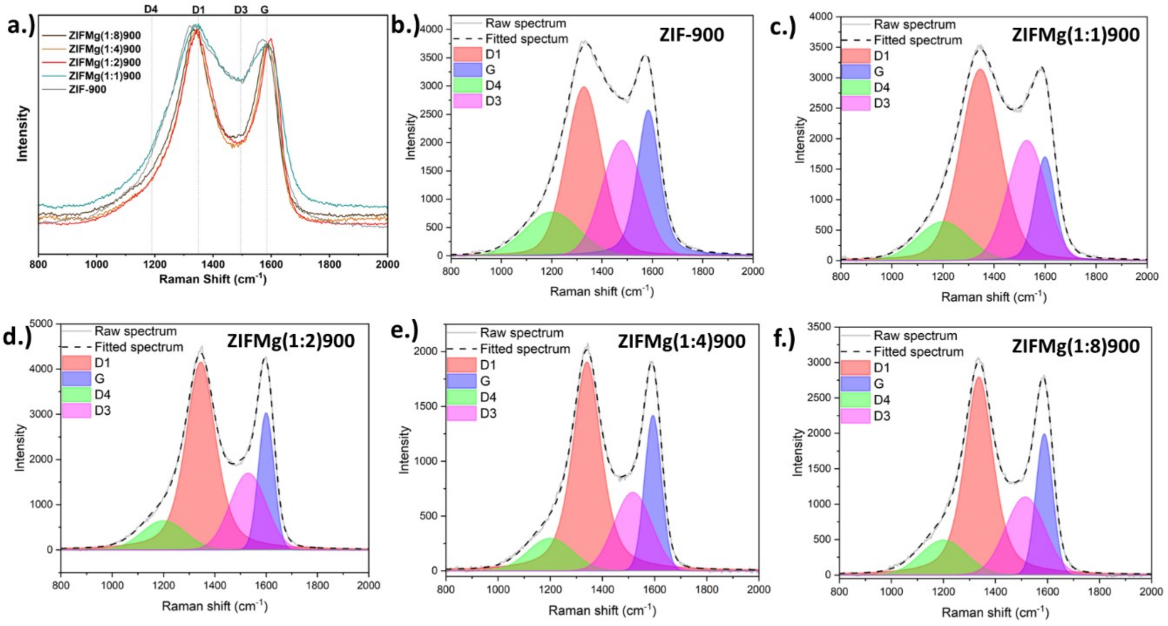

Figure S4. Raman spectra of a.) overlayed ZIF and Mg-ZIF derived catalysts with the positions of the carbon D and G bands indicated by vertical lines. Deconvolution of D and G bands in b.) ZIF-900 c.) ZIFMg(1:1)900 d.) ZIFMg(1:2)900 e.) ZIFMg(1:4)900 f.) ZIFMg(1:8)900.

Table S2. Parameters calculated from Raman deconvolution. The error relates to the standard deviation from nine repeated measurements, with the average value shown.

Sample	Intensity ratio (I_D/I_G)	Area ratio (A_D/A_G)	FWHM D band (cm^{-1})	FWHM G band (cm^{-1})	I_{D3}/I_G
**ZIF-900**	1.39 ± 0.10	2.76 ± 0.52	167 ± 8	96 ± 6	1.09 ± 0.22
**ZIFMg(1:1)900**	1.66 ± 0.16	3.03 ± 0.67	181 ± 5	96 ± 5	1.03 ± 0.11
**ZIFMg(1:2)900**	1.36 ± 0.02	3.25 ± 0.16	139 ± 1	71 ± 1	0.56 ± 0.02
**ZIFMg(1:4)900**	1.33 ± 0.05	2.98 ± 0.20	131 ± 6	76 ± 2	0.48 ± 0.04
**ZIFMg(1:8)900**	1.38 ± 0.04	3.02 ± 0.20	131 ± 3	77 ± 1	0.52 ± 0.02

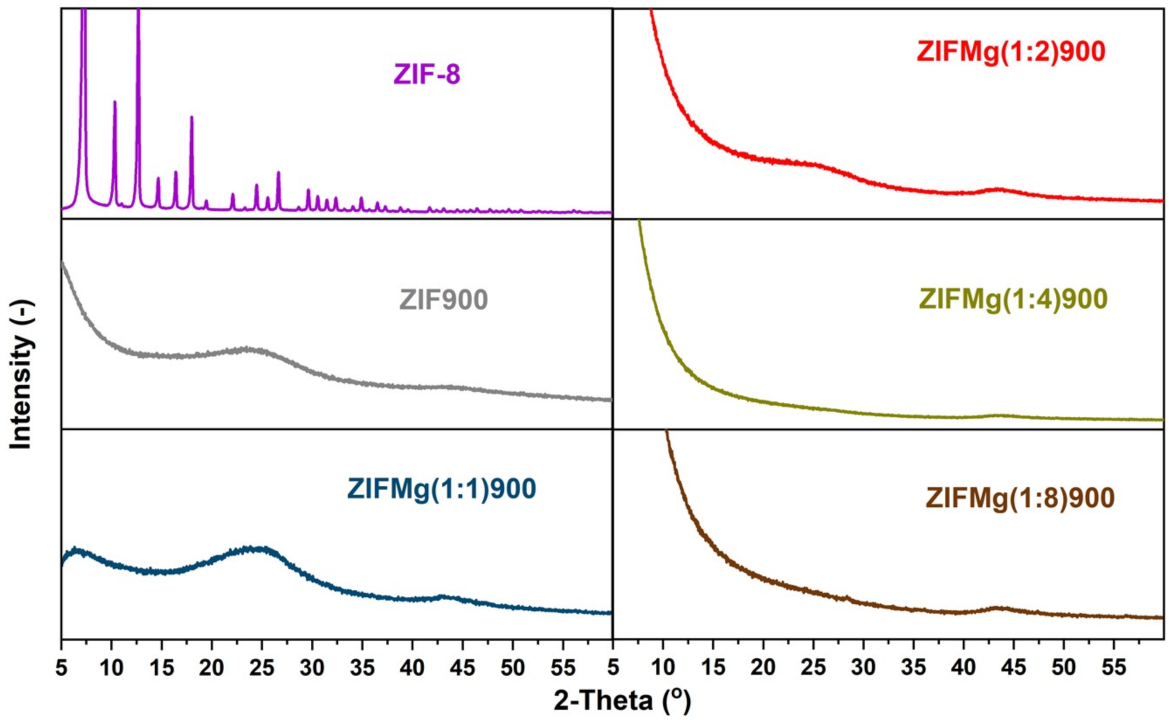

Figure S5. XRD of ZIF-8 and ZIF-Mg-derived catalyst powders.

Table S3. Specific surface area and pore volumes of ZIF-derived catalysts.

Sample	S_{BET} (m^2 g^{-1})	Micropore Volume, <1 nm (cm^3 g^{-1})	Micropore Volume, >1 nm (cm^3 g^{-1})	Mesopore Volume, <4 nm (cm^3 g^{-1})	Mesopore Volume, >4 nm (cm^3 g^{-1})
ZIF-8	1,170	0.168	0.368	---	---
ZIF900	568	0.171	0.055	0.019	0.032
ZIFMg(1:1)900	1,138	0.321	0.154	0.004	---
ZIFMg(1:2)900	2,254	0.488	0.381	0.057	0.013
ZIFMg(1:4)900	2,760	0.385	0.493	0.423	0.599
ZIFMg(1:8)900	3,548	0.383	0.631	0.841	0.455

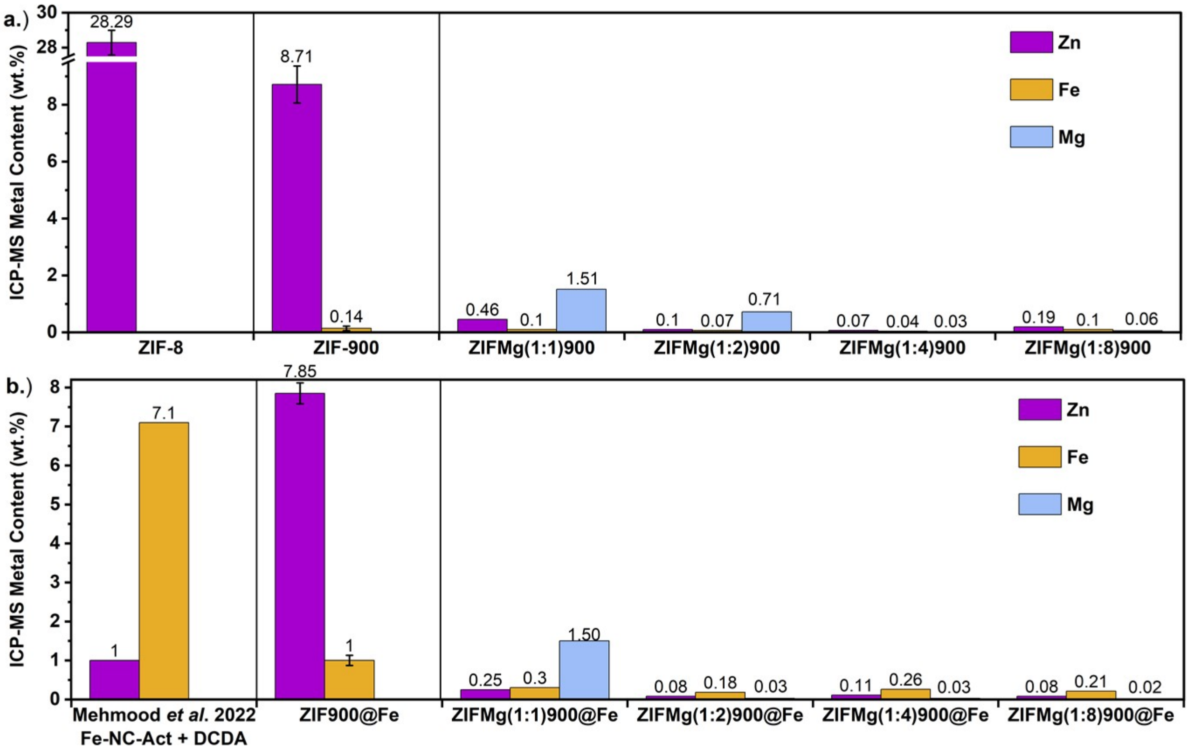

Figure S6. ICP-MS of ZIF-8 and its derived catalyst (a) following pyrolysis and (b) after Fe loading. Fe-N-C of Mehmood *et al.* was synthesised from the same commercial ZIF-8 (Basolite® Z1200) and methanol reflux Fe loading method and acid washing, but with 2 M H_2SO_4 reflux washing and an additional activation step with dicyandiamide (DCDA).[10] Error bars represent the error from two separate measurements.

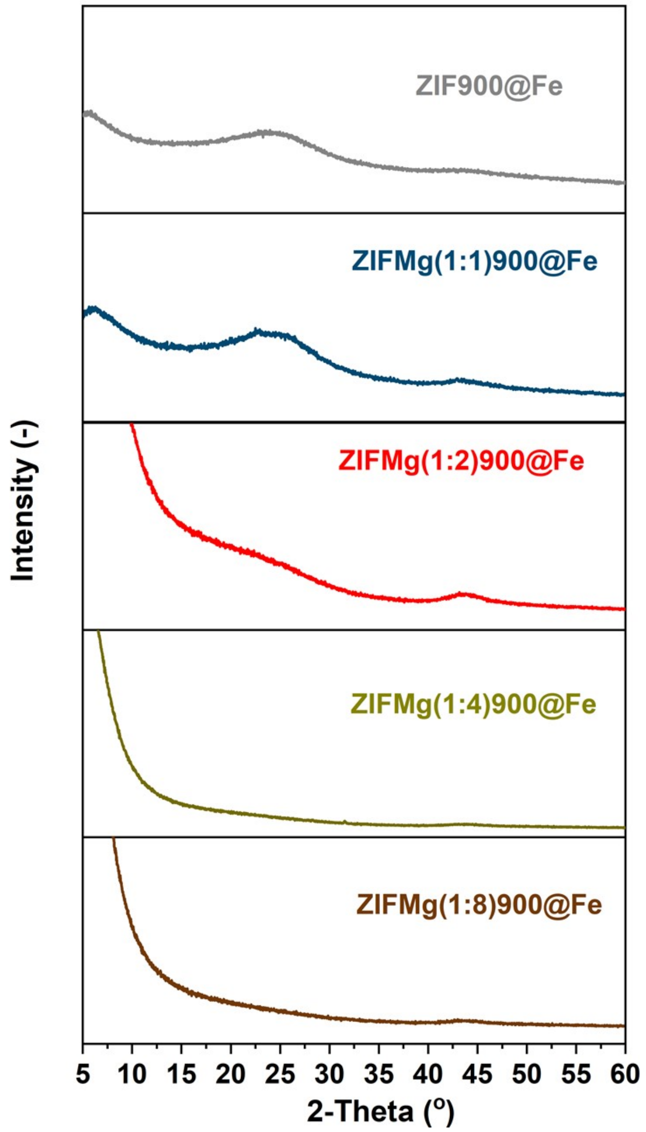

Figure S7. XRD of Fe-loaded ZIF and ZIF-Mg derived catalyst powders.

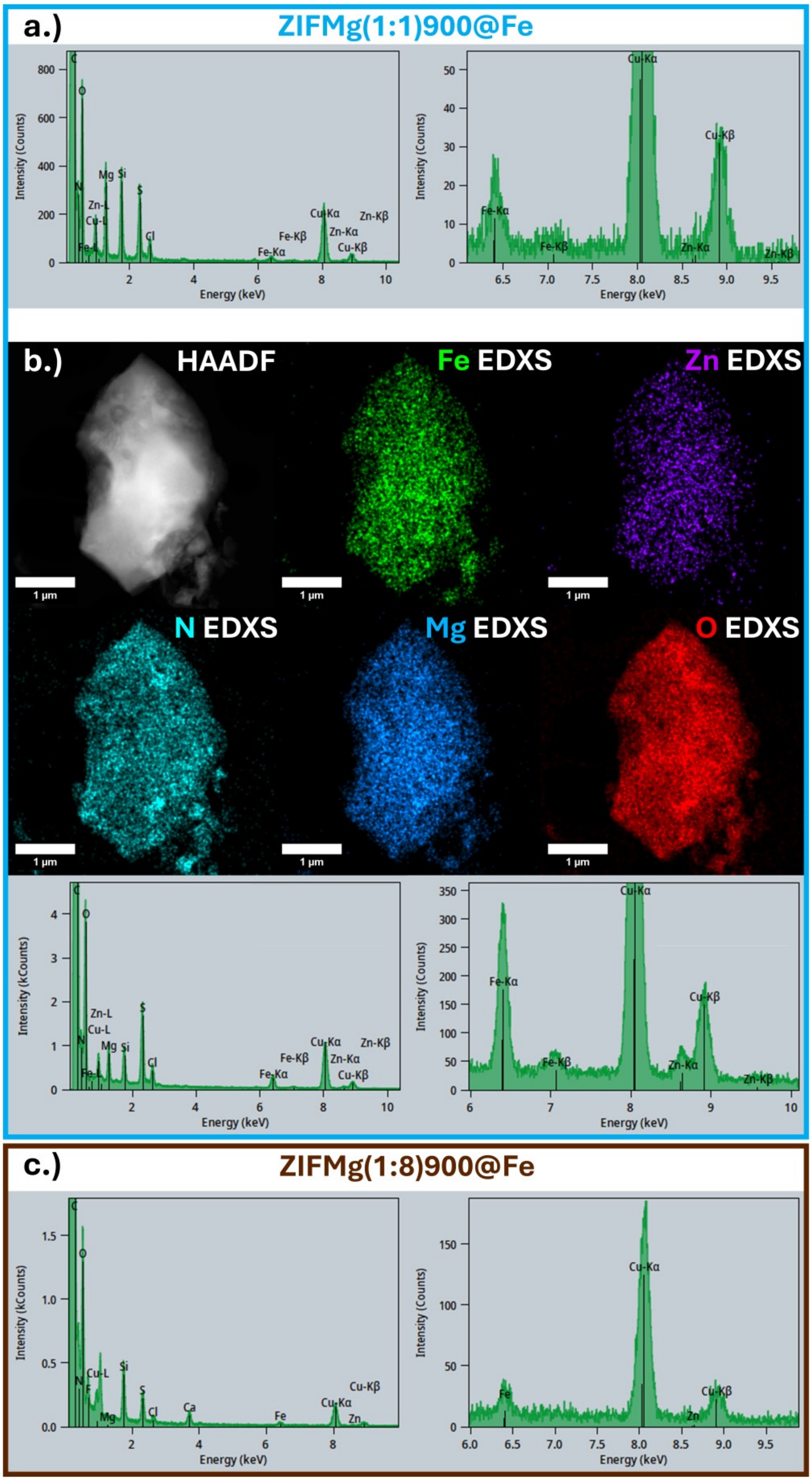

Figure S8. ZIFMg(1:1)900@Fe a.) Left: Summed EDXS spectrum from the elemental maps in Figure 2a, with right: enlarged Fe, Zn and Cu-K region. b.) HAADF-STEM image and EDXS maps of Fe, Zn, N, Mg and O with the summed EDXS spectrum shown below (left) together with an enlarged view of the Fe, Zn and Cu-K region (right). c.) ZIFMg(1:8)900@Fe. Left: Summed EDXS spectrum from the elemental maps in Figure 2d, with right: enlarged Fe, Zn and Cu-K region. Cu signals in all spectra are an artefact due to the Cu TEM support grid.

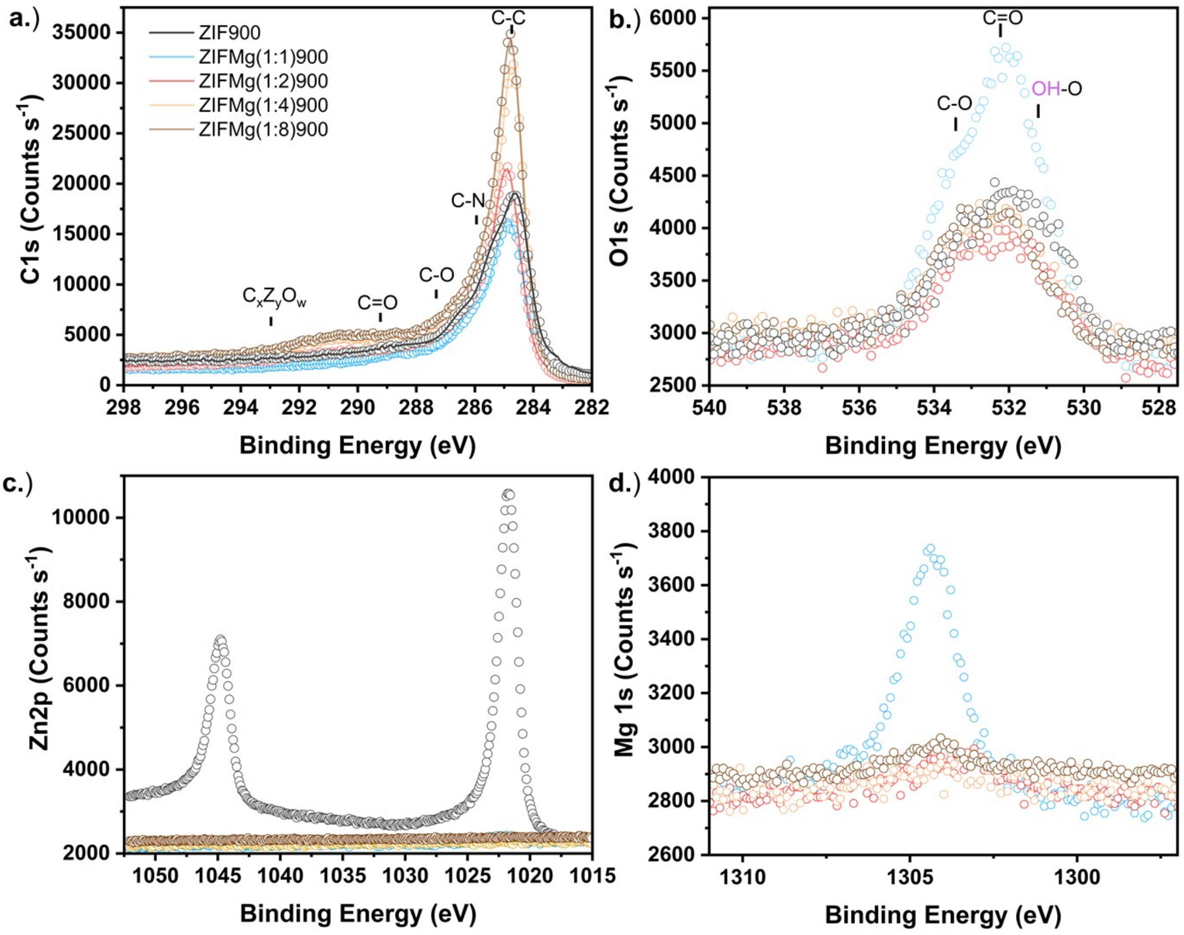

Figure S9. XPS overlays of ZIF-derived catalysts for a.) C1s b.) O1s c.) Zn2p d.) Mg1s.

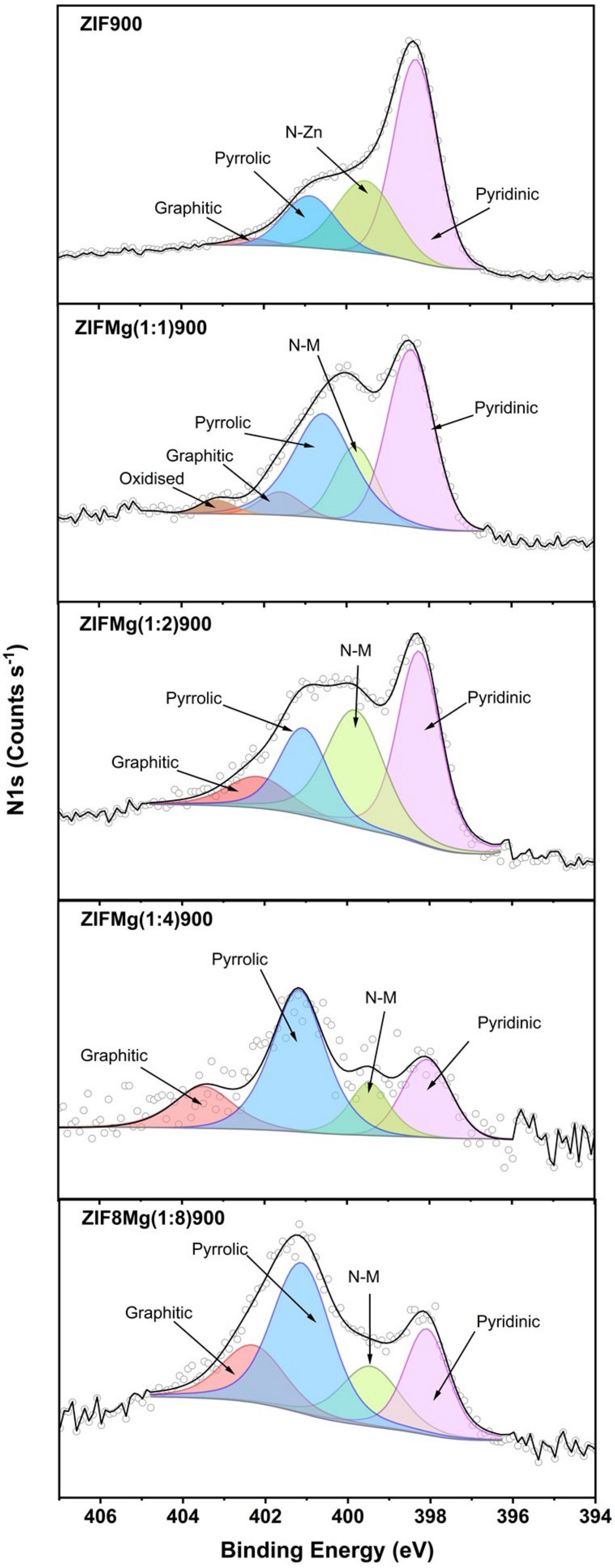

Figure S10. XPS of ZIF and ZIF-Mg derived catalysts a.) Fitted N species b.) N1s fittings. Circles represent raw XPS data and lines represent fitted spectra.

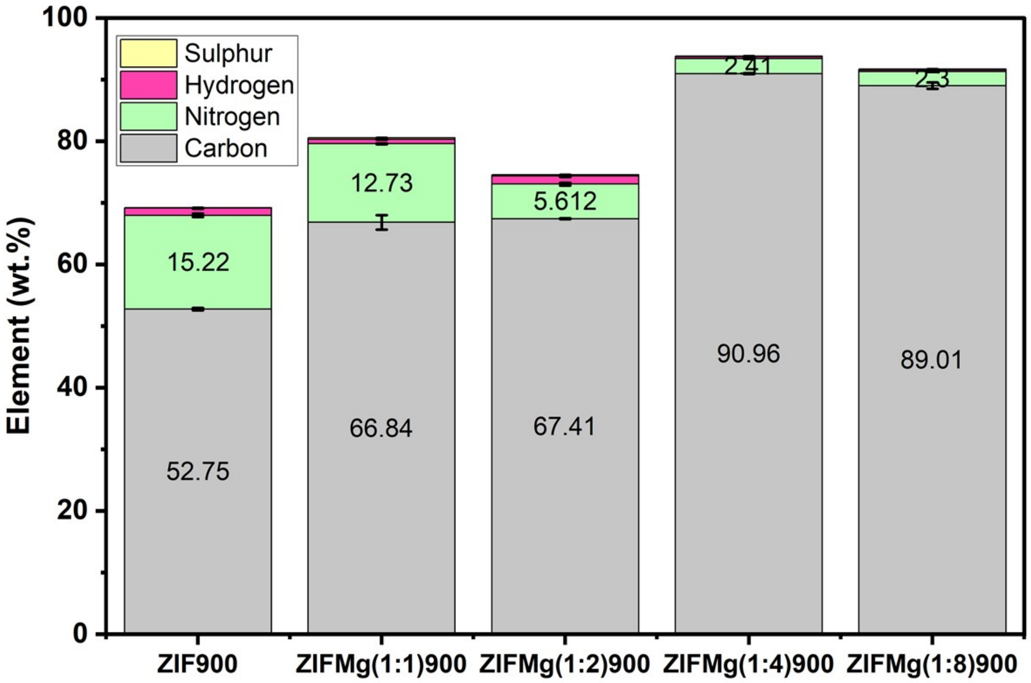

Figure S11. Elemental CHNS analysis for ZIF-Mg derived catalysts. Error bars represent deviation from two measurements.

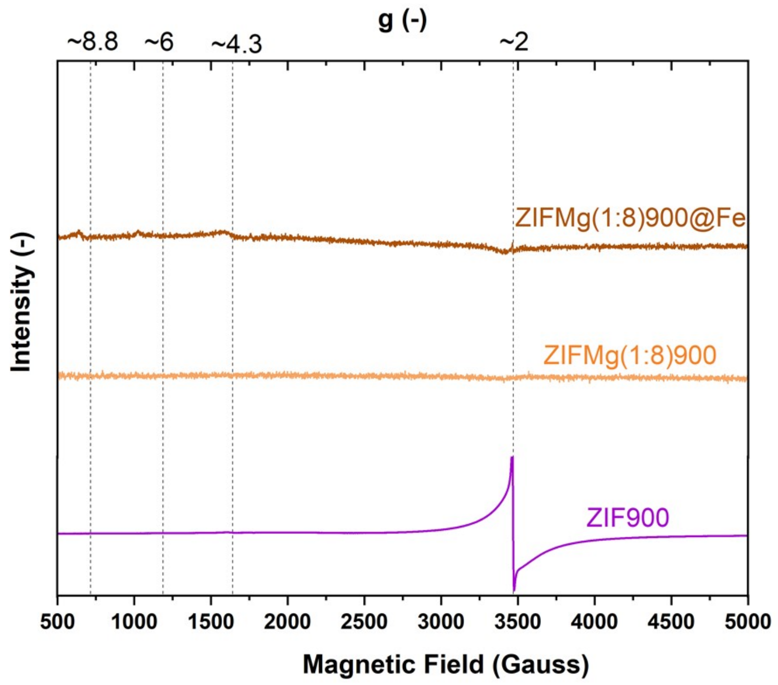

Figure S12. Cryo (5 K) X-band EPR spectra for ZIF900, ZIFMg(1:8)900, ZIFMg(1:8)900@Fe.

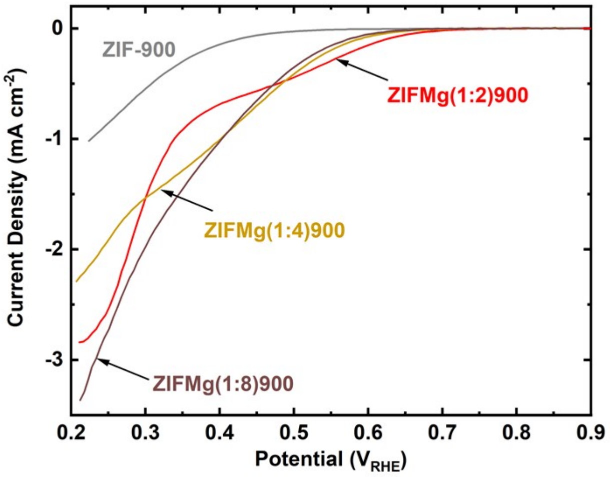

Figure S13. 3rd scan of cyclic voltammogram in cathodic direction with RDE at 1,600 rpm in O_2-saturated 0.1 M $HClO_4$ from 0.90-0.20 V_{RHE} at 10 mV s^{-1} with 0.26 $mg_{M\text{-}N\text{-}C}$ cm^{-2} for fresh ZIF900 and ZIFMg(1:X)900 catalyst. Measurements under equivalent N_2-saturated conditions were subtracted from those measured under O_2-saturated.

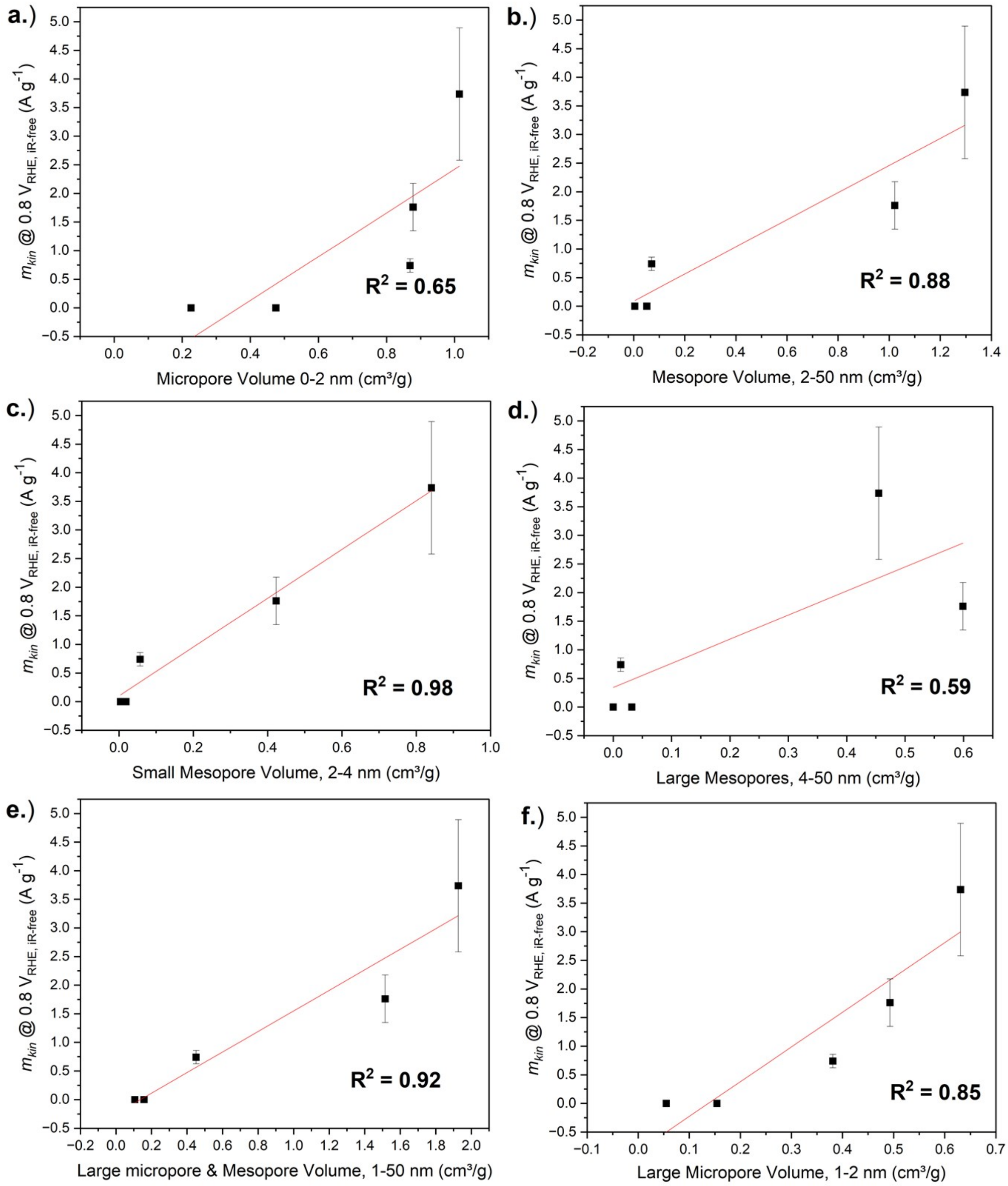

Figure S14. Correlation between kinetic mass activity at 0.8 $V_{RHE,iR-free}$ and a.) Micropore volume, 0-2 nm. b.) Mesopore volume, 2-50 nm. c.) Small Mesopore volume, 2-4 nm. d.) Large Mesopore volume, >4 nm. e.) Large Micropore and Mesopore Volume, 1-50 nm. f.) Large Micropore Volume, 1-2 nm. Error bars represents difference from average for two repeat measurements.

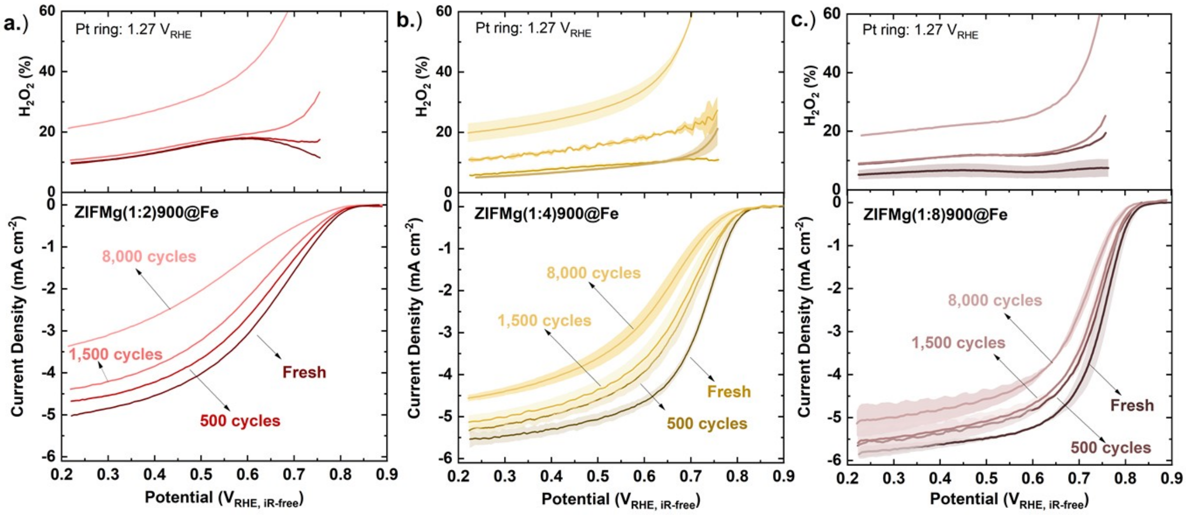

Figure S15. 3rd scan of cyclic voltammogram in cathodic direction with RRDE at 1,600 rpm in O_2-saturated 0.1 M $HClO_4$ from 0.90-0.20 V_{RHE} at 10 mV s^{-1} with 0.26 $mg_{Fe\text{-}N\text{-}C}$ cm^{-2} over AST cycles (0.80-0.40 V_{RHE} at 100 mV s^{-1} under O_2-saturation at 1,600 rpm) for a.) ZIFMg(1:2)900@Fe b.) ZIFMg(1:4)900@Fe c.) ZIFMg(1:8)900@Fe. Measurements under equivalent N_2-saturated conditions were subtracted from those measured under O_2-saturated.

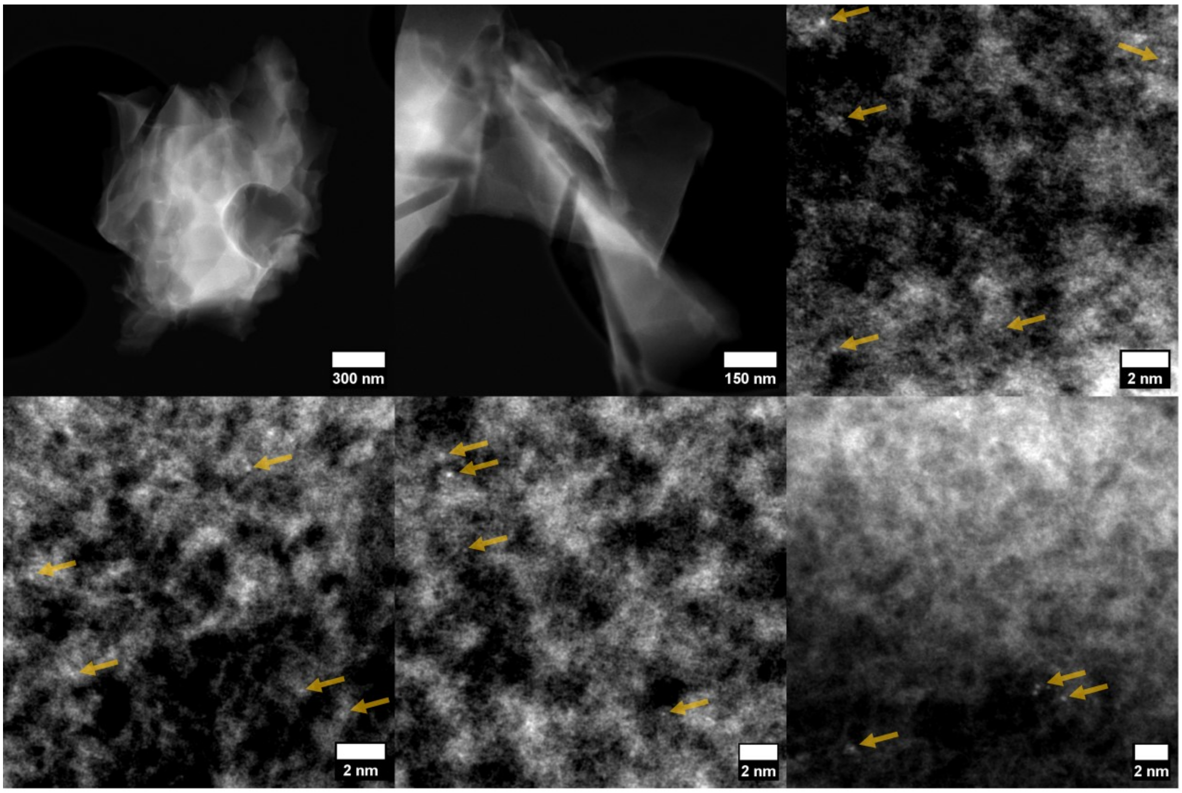

Figure S16. HAADF-STEM of ZIFMg(1:8)900@Fe post 8000 AST cycles (0.80-0.40 V_{RHE} at 100 mV s^{-1} under O_2-saturation in 0.1 M $HClO_4$ at 1,600 rpm). Yellow arrows highlight possible metal atoms.

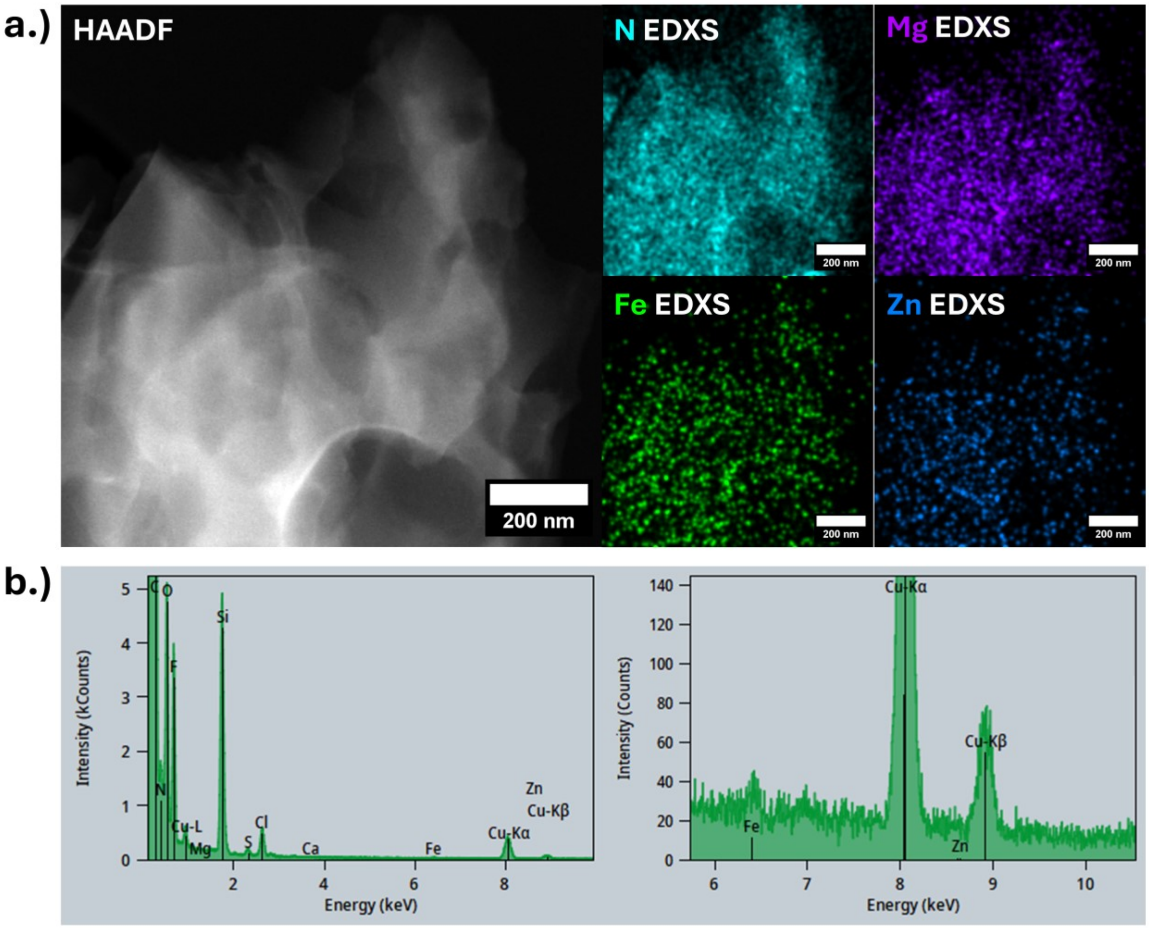

Figure S17. ZIFMg(1:8)900@Fe post 8000 AST cycles (0.80-0.40 V_{RHE} at 100 mV s^{-1} under O_2-saturation in 0.1 M $HClO_4$ at 1,600 rpm) a.) HAADF-STEM image and corresponding EDXS mapping of Fe, Zn, N, Mg and F. b.) Left: summed EDXS spectrum with right: enlarged Fe, Zn and Cu-K region. The Cu signal is due to the Cu TEM support grid.

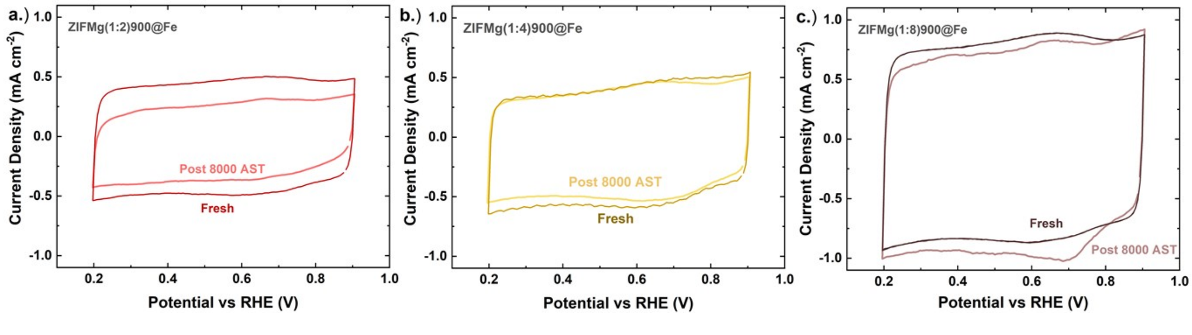

Figure S18. 10th scan cyclic voltammogram in N_2-saturated 0.1 M $HClO_4$ over 0.9-0.20 V_{RHE} at 50 mV s^{-1} with 0.26 $mg_{Fe\text{-}N\text{-}C}$ cm^{-2} for fresh and post 8000 AST cycles (0.80-0.40 V_{RHE} at 100 mV s^{-1} under O_2-saturation at 1,600 rpm) a.) ZIFMg(1:2)900@Fe b.) ZIFMg(1:4)900@Fe c.) ZIFMg(1:8)900@Fe.

References

[1] J. Barrio, A. Pedersen, S. Ch. Sarma, A. Bagger, M. Gong, S. Favero, C.-X. Zhao, R. Garcia-Serres, A. Y. Li, Q. Zhang, F. Jaouen, F. Maillard, A. Kucernak, I. E. L. Stephens, M.-M. Titirici, *Adv. Mater.* **2023**, *35*, 2211022.

[2] J. Barrio, A. Pedersen, J. Feng, S. Ch. Sarma, M. Wang, A. Y. Li, H. Yadegari, H. Luo, M. P. Ryan, M.-M. Titirici, Ifan. E. L. Stephens, *J. Mater. Chem. A* **2022**, *10*, 6023.

[3] A. Mehmood, J. Pampel, G. Ali, H. Y. Ha, F. Ruiz-Zepeda, T.-P. Fellinger, *Adv. Energy Mater.* **2018**, *8*, 1701771.

[4] L. Castanheira, W. O. Silva, F. H. B. Lima, A. Crisci, L. Dubau, F. Maillard, *ACS Catal.* **2015**, *5*, 2184.

[5] T. Jawhari, A. Roid, J. Casado, *Carbon* **1995**, *33*, 1561.

[6] A. Sadezky, H. Muckenhuber, H. Grothe, R. Niessner, U. Pöschl, *Carbon* **2005**, *43*, 1731.

[7] J. Rouquerol, P. Llewellyn, F. Rouquerol, in *Stud. Surf. Sci. Catal.*, Elsevier, **2007**, pp. 49–56.

[8] J. Jagiello, J. P. Olivier, *Adsorption* **2013**, *19*, 777.

[9] R. Zhou, Y. Zheng, M. Jaroniec, S.-Z. Qiao, *ACS Catal.* **2016**, *6*, 4720.

[10] A. Mehmood, M. Gong, F. Jaouen, A. Roy, A. Zitolo, A. Khan, M. Sougrati, M. Primbs, A. M. Bonastre, D. Fongalland, G. Drazic, P. Strasser, A. Kucernak, *Nat. Catal.* **2022**, *5*, 311.
